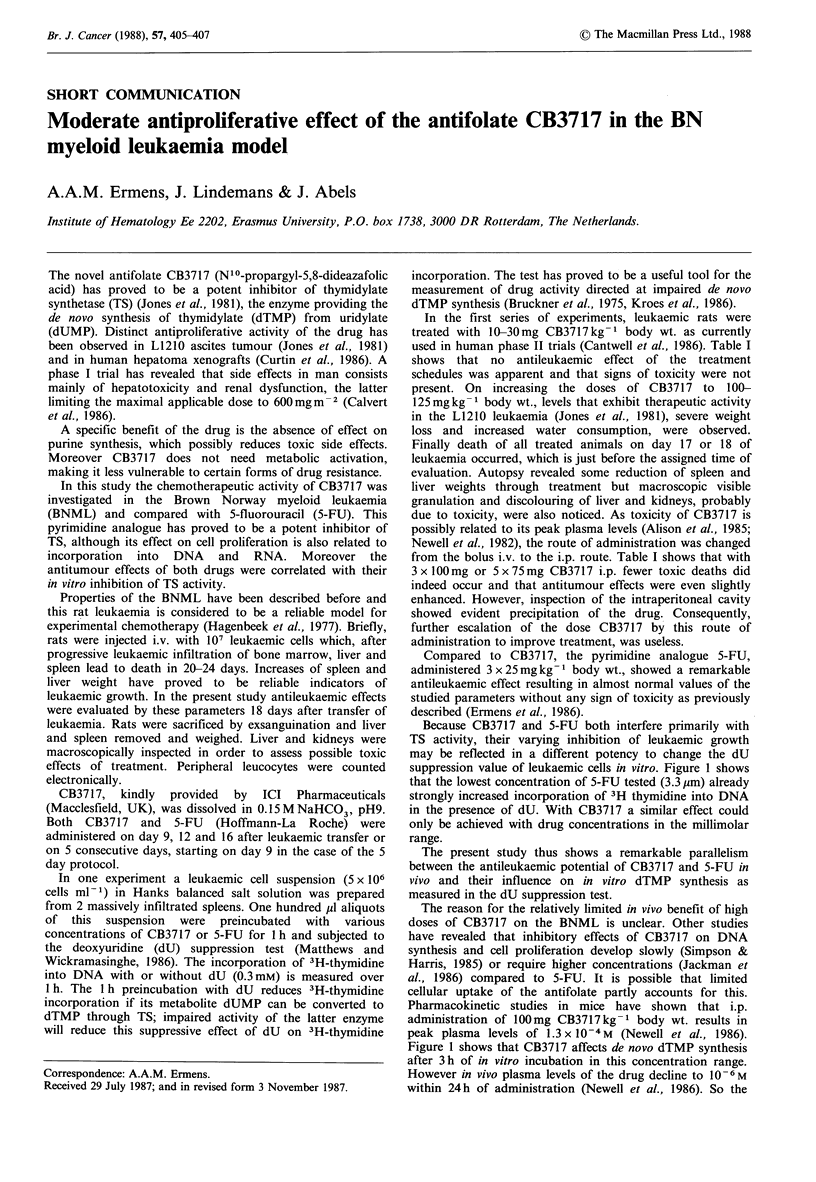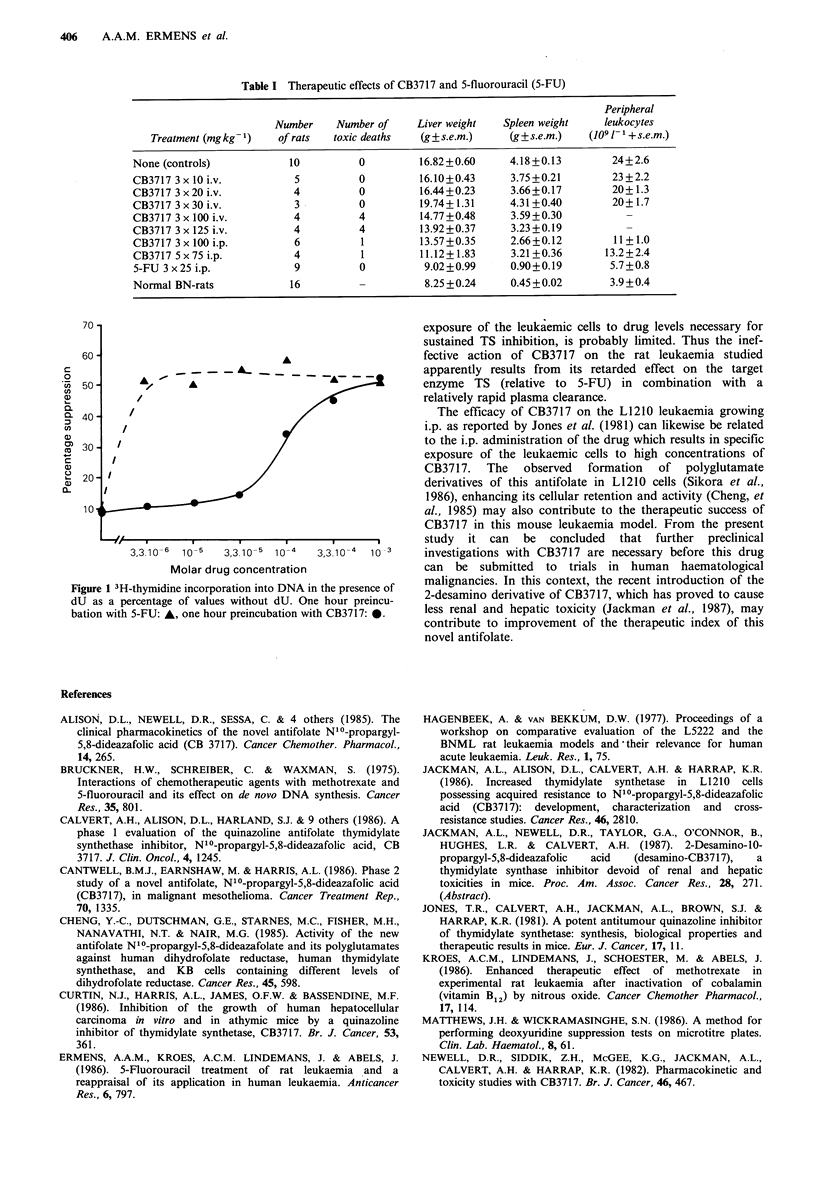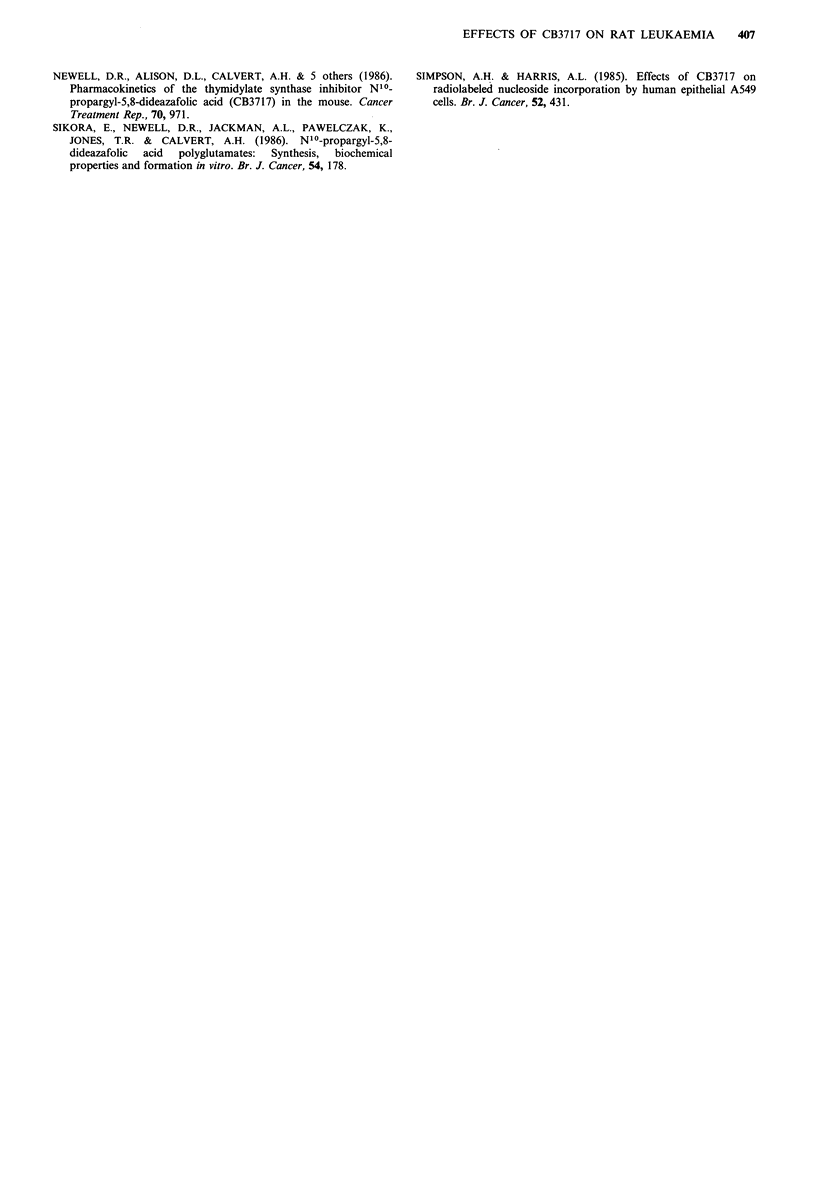# Moderate antiproliferative effect of the antifolate CB3717 in the BN myeloid leukaemia model.

**DOI:** 10.1038/bjc.1988.91

**Published:** 1988-04

**Authors:** A. A. Ermens, J. Lindemans, J. Abels

**Affiliations:** Institute of Hematology Ee 2202, Erasmus University, Rotterdam, The Netherlands.


*Br. J. Cancer* (1988), **57**, 405–407 © The Macmillan Press Ltd., 1988

SHORT COMMUNICATION

### Moderate antiproliferative effect of the antifolate CB3717 in the BN myeloid leukaemia model

A.A.M. Ermens, J. Lindemans & J. Abels

*Institute of Hematology Ee 2202, Erasmus University, P.O. box 1738, 3000 DR Rotterdam, The Netherlands.*

The novel antifolate CB3717 ($N^{10}$-propargyl-5,8-dideazafolic acid) has proved to be a potent inhibitor of thymidylate synthetase (TS) (Jones *et al.*, 1981), the enzyme providing the *de novo* synthesis of thymidylate (dTMP) from uridylate (dUMP). Distinct antiproliferative activity of the drug has been observed in L1210 ascites tumour (Jones *et al.*, 1981) and in human hepatoma xenografts (Curtin *et al.*, 1986). A phase I trial has revealed that side effects in man consists mainly of hepatotoxicity and renal dysfunction, the latter limiting the maximal applicable dose to $600 \, mg \, m^{-2}$ (Calvert *et al.*, 1986).

A specific benefit of the drug is the absence of effect on purine synthesis, which possibly reduces toxic side effects. Moreover CB3717 does not need metabolic activation, making it less vulnerable to certain forms of drug resistance.

In this study the chemotherapeutic activity of CB3717 was investigated in the Brown Norway myeloid leukaemia (BNML) and compared with 5-fluorouracil (5-FU). This pyrimidine analogue has proved to be a potent inhibitor of TS, although its effect on cell proliferation is also related to incorporation into DNA and RNA. Moreover the antitumour effects of both drugs were correlated with their *in vitro* inhibition of TS activity.

Properties of the BNML have been described before and this rat leukaemia is considered to be a reliable model for experimental chemotherapy (Hagenbeek *et al.*, 1977). Briefly, rats were injected i.v. with $10^7$ leukaemic cells which, after progressive leukaemic infiltration of bone marrow, liver and spleen lead to death in 20–24 days. Increases of spleen and liver weight have proved to be reliable indicators of leukaemic growth. In the present study antileukaemic effects were evaluated by these parameters 18 days after transfer of leukaemia. Rats were sacrificed by exsanguination and liver and spleen removed and weighed. Liver and kidneys were macroscopically inspected in order to assess possible toxic effects of treatment. Peripheral leucocytes were counted electronically.

CB3717, kindly provided by ICI Pharmaceuticals (Macclesfield, UK), was dissolved in $0.15 \, M \, NaHCO_3$, pH9. Both CB3717 and 5-FU (Hoffmann-La Roche) were administered on day 9, 12 and 16 after leukaemic transfer or on 5 consecutive days, starting on day 9 in the case of the 5 day protocol.

In one experiment a leukaemic cell suspension ($5 \times 10^6$ cells $ml^{-1}$) in Hanks balanced salt solution was prepared from 2 massively infiltrated spleens. One hundred $\mu l$ aliquots of this suspension were preincubated with various concentrations of CB3717 or 5-FU for 1 h and subjected to the deoxyuridine (dU) suppression test (Matthews and Wickramasinghe, 1986). The incorporation of $^3H$-thymidine into DNA with or without dU (0.3 mM) is measured over 1 h. The 1 h preincubation with dU reduces $^3H$-thymidine incorporation if its metabolite dUMP can be converted to dTMP through TS; impaired activity of the latter enzyme will reduce this suppressive effect of dU on $^3H$-thymidine incorporation. The test has proved to be a useful tool for the measurement of drug activity directed at impaired *de novo* dTMP synthesis (Bruckner *et al.*, 1975, Kroes *et al.*, 1986).

In the first series of experiments, leukaemic rats were treated with 10–30 mg CB3717 $kg^{-1}$ body wt. as currently used in human phase II trials (Cantwell *et al.*, 1986). Table I shows that no antileukaemic effect of the treatment schedules was apparent and that signs of toxicity were not present. On increasing the doses of CB3717 to 100–125 mg $kg^{-1}$ body wt., levels that exhibit therapeutic activity in the L1210 leukaemia (Jones *et al.*, 1981), severe weight loss and increased water consumption, were observed. Finally death of all treated animals on day 17 or 18 of leukaemia occurred, which is just before the assigned time of evaluation. Autopsy revealed some reduction of spleen and liver weights through treatment but macroscopic visible granulation and discolouring of liver and kidneys, probably due to toxicity, were also noticed. As toxicity of CB3717 is possibly related to its peak plasma levels (Alison *et al.*, 1985; Newell *et al.*, 1982), the route of administration was changed from the bolus i.v. to the i.p. route. Table I shows that with $3 \times 100$ mg or $5 \times 75$ mg CB3717 i.p. fewer toxic deaths did indeed occur and that antitumour effects were even slightly enhanced. However, inspection of the intraperitoneal cavity showed evident precipitation of the drug. Consequently, further escalation of the dose CB3717 by this route of administration to improve treatment, was useless.

Compared to CB3717, the pyrimidine analogue 5-FU, administered $3 \times 25$ mg $kg^{-1}$ body wt., showed a remarkable antileukaemic effect resulting in almost normal values of the studied parameters without any sign of toxicity as previously described (Ermens *et al.*, 1986).

Because CB3717 and 5-FU both interfere primarily with TS activity, their varying inhibition of leukaemic growth may be reflected in a different potency to change the dU suppression value of leukaemic cells *in vitro*. Figure 1 shows that the lowest concentration of 5-FU tested (3.3 $\mu$m) already strongly increased incorporation of $^3H$ thymidine into DNA in the presence of dU. With CB3717 a similar effect could only be achieved with drug concentrations in the millimolar range.

The present study thus shows a remarkable parallelism between the antileukaemic potential of CB3717 and 5-FU *in vivo* and their influence on *in vitro* dTMP synthesis as measured in the dU suppression test.

The reason for the relatively limited *in vivo* benefit of high doses of CB3717 on the BNML is unclear. Other studies have revealed that inhibitory effects of CB3717 on DNA synthesis and cell proliferation develop slowly (Simpson & Harris, 1985) or require higher concentrations (Jackman *et al.*, 1986) compared to 5-FU. It is possible that limited cellular uptake of the antifolate partly accounts for this. Pharmacokinetic studies in mice have shown that i.p. administration of 100 mg CB3717 $kg^{-1}$ body wt. results in peak plasma levels of $1.3 \times 10^{-4}$ M (Newell *et al.*, 1986). Figure 1 shows that CB3717 affects *de novo* dTMP synthesis after 3 h of *in vitro* incubation in this concentration range. However *in vivo* plasma levels of the drug decline to $10^{-6}$ M within 24 h of administration (Newell *et al.*, 1986). So the

Correspondence: A.A.M. Ermens.
Received 29 July 1987; and in revised form 3 November 1987.

**Table I** Therapeutic effects of CB3717 and 5-fluorouracil (5-FU)

| *Treatment* (*mg kg*$^{-1}$) | *Number of rats* | *Number of toxic deaths* | *Liver weight* (*g* ± *s.e.m.*) | *Spleen weight* (*g* ± *s.e.m.*) | *Peripheral leukocytes* ($10^9\ l^{-1}$ + *s.e.m.*) |
|---|---|---|---|---|---|
| None (controls) | 10 | 0 | 16.82±0.60 | 4.18±0.13 | 24±2.6 |
| CB3717 3×10 i.v. | 5 | 0 | 16.10±0.43 | 3.75±0.21 | 23±2.2 |
| CB3717 3×20 i.v. | 4 | 0 | 16.44±0.23 | 3.66±0.17 | 20±1.3 |
| CB3717 3×30 i.v. | 3 | 0 | 19.74±1.31 | 4.31±0.40 | 20±1.7 |
| CB3717 3×100 i.v. | 4 | 4 | 14.77±0.48 | 3.59±0.30 | – |
| CB3717 3×125 i.v. | 4 | 4 | 13.92±0.37 | 3.23±0.19 | – |
| CB3717 3×100 i.p. | 6 | 1 | 13.57±0.35 | 2.66±0.12 | 11±1.0 |
| CB3717 5×75 i.p. | 4 | 1 | 11.12±1.83 | 3.21±0.36 | 13.2±2.4 |
| 5-FU 3×25 i.p. | 9 | 0 | 9.02±0.99 | 0.90±0.19 | 5.7±0.8 |
| Normal BN-rats | 16 | – | 8.25±0.24 | 0.45±0.02 | 3.9±0.4 |

**Figure 1** ³H-thymidine incorporation into DNA in the presence of dU as a percentage of values without dU. One hour preincubation with 5-FU: ▲, one hour preincubation with CB3717: ●.

exposure of the leukaemic cells to drug levels necessary for sustained TS inhibition, is probably limited. Thus the ineffective action of CB3717 on the rat leukaemia studied apparently results from its retarded effect on the target enzyme TS (relative to 5-FU) in combination with a relatively rapid plasma clearance.

The efficacy of CB3717 on the L1210 leukaemia growing i.p. as reported by Jones *et al.* (1981) can likewise be related to the i.p. administration of the drug which results in specific exposure of the leukaemic cells to high concentrations of CB3717. The observed formation of polyglutamate derivatives of this antifolate in L1210 cells (Sikora *et al.*, 1986), enhancing its cellular retention and activity (Cheng, *et al.*, 1985) may also contribute to the therapeutic success of CB3717 in this mouse leukaemia model. From the present study it can be concluded that further preclinical investigations with CB3717 are necessary before this drug can be submitted to trials in human haematological malignancies. In this context, the recent introduction of the 2-desamino derivative of CB3717, which has proved to cause less renal and hepatic toxicity (Jackman *et al.*, 1987), may contribute to improvement of the therapeutic index of this novel antifolate.

### References

ALISON, D.L., NEWELL, D.R., SESSA, C. & 4 others (1985). The clinical pharmacokinetics of the novel antifolate N¹⁰-propargyl-5,8-dideazafolic acid (CB 3717). *Cancer Chemother. Pharmacol.*, **14**, 265.

BRUCKNER, H.W., SCHREIBER, C. & WAXMAN, S. (1975). Interactions of chemotherapeutic agents with methotrexate and 5-fluorouracil and its effect on *de novo* DNA synthesis. *Cancer Res.*, **35**, 801.

CALVERT, A.H., ALISON, D.L., HARLAND, S.J. & 9 others (1986). A phase 1 evaluation of the quinazoline antifolate thymidylate synthethase inhibitor, N¹⁰-propargyl-5,8-dideazafolic acid, CB 3717. *J. Clin. Oncol.*, **4**, 1245.

CANTWELL, B.M.J., EARNSHAW, M. & HARRIS, A.L. (1986). Phase 2 study of a novel antifolate, N¹⁰-propargyl-5,8-dideazafolic acid (CB3717), in malignant mesothelioma. *Cancer Treatment Rep.*, **70**, 1335.

CHENG, Y.-C., DUTSCHMAN, G.E., STARNES, M.C., FISHER, M.H., NANAVATHI, N.T. & NAIR, M.G. (1985). Activity of the new antifolate N¹⁰-propargyl-5,8-dideazafolate and its polyglutamates against human dihydrofolate reductase, human thymidylate synthethase, and KB cells containing different levels of dihydrofolate reductase. *Cancer Res.*, **45**, 598.

CURTIN, N.J., HARRIS, A.L., JAMES, O.F.W. & BASSENDINE, M.F. (1986). Inhibition of the growth of human hepatocellular carcinoma *in vitro* and in athymic mice by a quinazoline inhibitor of thymidylate synthetase, CB3717. *Br. J. Cancer*, **53**, 361.

ERMENS, A.A.M., KROES, A.C.M. LINDEMANS, J. & ABELS, J. (1986). 5-Fluorouracil treatment of rat leukaemia and a reappraisal of its application in human leukaemia. *Anticancer Res.*, **6**, 797.

HAGENBEEK, A. & VAN BEKKUM, D.W. (1977). Proceedings of a workshop on comparative evaluation of the L5222 and the BNML rat leukaemia models and their relevance for human acute leukaemia. *Leuk. Res.*, **1**, 75.

JACKMAN, A.L., ALISON, D.L., CALVERT, A.H. & HARRAP, K.R. (1986). Increased thymidylate synthetase in L1210 cells possessing acquired resistance to N¹⁰-propargyl-5,8-dideazafolic acid (CB3717): development, characterization and cross-resistance studies. *Cancer Res.*, **46**, 2810.

JACKMAN, A.L., NEWELL, D.R., TAYLOR, G.A., O'CONNOR, B., HUGHES, L.R. & CALVERT, A.H. (1987). 2-Desamino-10-propargyl-5,8-dideazafolic acid (desamino-CB3717), a thymidylate synthase inhibitor devoid of renal and hepatic toxicities in mice. *Proc. Am. Assoc. Cancer Res.*, **28**, 271. (*Abstract*).

JONES, T.R., CALVERT, A.H., JACKMAN, A.L., BROWN, S.J. & HARRAP, K.R. (1981). A potent antitumour quinazoline inhibitor of thymidylate synthetase: synthesis, biological properties and therapeutic results in mice. *Eur. J. Cancer*, **17**, 11.

KROES, A.C.M., LINDEMANS, J., SCHOESTER, M. & ABELS, J. (1986). Enhanced therapeutic effect of methotrexate in experimental rat leukaemia after inactivation of cobalamin (vitamin $B_{12}$) by nitrous oxide. *Cancer Chemother Pharmacol.*, **17**, 114.

MATTHEWS, J.H. & WICKRAMASINGHE, S.N. (1986). A method for performing deoxyuridine suppression tests on microtitre plates. *Clin. Lab. Haematol.*, **8**, 61.

NEWELL, D.R., SIDDIK, Z.H., McGEE, K.G., JACKMAN, A.L., CALVERT, A.H. & HARRAP, K.R. (1982). Pharmacokinetic and toxicity studies with CB3717. *Br. J. Cancer*, **46**, 467.

NEWELL, D.R., ALISON, D.L., CALVERT, A.H. & 5 others (1986). Pharmacokinetics of the thymidylate synthase inhibitor $N^{10}$-propargyl-5,8-dideazafolic acid (CB3717) in the mouse. *Cancer Treatment Rep.*, **70**, 971.

SIKORA, E., NEWELL, D.R., JACKMAN, A.L., PAWELCZAK, K., JONES, T.R. & CALVERT, A.H. (1986). $N^{10}$-propargyl-5,8-dideazafolic acid polyglutamates: Synthesis, biochemical properties and formation *in vitro*. *Br. J. Cancer*, **54**, 178.

SIMPSON, A.H. & HARRIS, A.L. (1985). Effects of CB3717 on radiolabeled nucleoside incorporation by human epithelial A549 cells. *Br. J. Cancer*, **52**, 431.